# Do Amelogenin Peptide-Based Nanohydrogels Prevent Artificially Induced White Spot Lesions?

**DOI:** 10.3290/j.jad.c_2843

**Published:** 2026-10-21

**Authors:** Maryam Shakir, Harraa S. Mohammed-Salih, Falah H. Hussein, Jameel R. Al-Obaidi

**Affiliations:** a Maryam Shakir PhD Researcher, Orthodontic Department, College of Dentistry, University of Baghdad, Baghdad, Iraq; Orthodontic Department, College of Dentistry, University of Al-Ameed, Karbala, Iraq. Study design, conducting the study, analyzing the data, writing and reviewing the manuscript.; b Harraa S. Mohammed-Salih Dentist, Orthodontic Department, College of Dentistry, University of Baghdad, Baghdad, Iraq. Study design, conducting the study, analyzing the data, writing and reviewing the manuscript.; c Falah H. Hussein PhD in Chemistry, College of Pharmacy, University of Babylon, Babylon, Iraq. Study design, conducting the study, analyzing the data, writing and reviewing the manuscript.; d Jameel R. Al-Obaidi PhD in Molecular Biology, Department of Biology, Faculty of Science and Mathematics, Universiti Pendidikan Sultan Idris, Tanjong Malim, Perak, Malaysia; Applied Science Research Center, Applied Science Private University, Amman, Jordan. Study design, conducting the study, analyzing the data, writing and reviewing the manuscript.

**Keywords:** amelogenin peptide, chitosan, enamel remineralization, nanohydrogel

## Abstract

**Purpose:**

As one of the most undesirable side effects of fixed orthodontic treatment, the occurrence of white spot lesions should be prevented. In the present study, amelogenin peptide combined with hydroxyapatite nanoparticles within chitosan hydrogels was formulated to prevent enamel demineralization.

**Methods and Materials:**

Three groups were evaluated for the evaluation of preventive effects. The first group received no treatment and was considered a control group, while the other groups were the experimental groups with the intervention. The first experimental group was treated with chitosan-based hydrogel including the peptide, while the other group was treated with the application of equal amounts of chitosan-based hydrogels loaded with either peptide or hydroxyapatite. pH cycling was carried out for two weeks. The preventive potential was evaluated through the estimation of enamel microhardness, fluorescence, and X-ray diffraction. The surface characteristics of sound and treated enamel were evaluated by scanning electron microscopy using different magnifications, and the chemical compositions were analyzed by an energy-dispersive X-ray spectroscopic analyzer.

**Results:**

The results revealed the preventive potential of amelogenin peptide combined with hydroxyapatite through the preservation and improvement of appearance, and mechanical and chemical characteristics of the enamel surface throughout pH cycling.

**Conclusion:**

This study concludes that the amelogenin peptide combined with hydroxyapatite nanoparticles within chitosan hydrogels provides a promising preventive strategy.

The demand for improved facial and smile esthetics with modern living styles has significantly increased, with a noticeable elevation in the number of people among different age groups who are seeking orthodontic treatment.^6,20
^ Among the various types of orthodontic appliances that are available nowadays, fixed orthodontic braces are still the most reliable and the most commonly used for efficient treatment despite their iatrogenic effects.^30^


Different types of bonded attachments included in fixed appliances impose stagnation areas for plaque accumulation that may not be removed easily through natural and mechanical cleaning mechanisms.^9^ After plaque accumulation, the acidogenic bacteria in plaque, most notably *Streptococcus mutans*, which are involved in the plaque, will diffuse acids that cause subsurface enamel demineralization.^52^ The area of demineralization will reflect light differently when compared with the adjacent sound enamel. The result will be white opaque spots named white spot lesions (WSLs).^1,39
^ The WSLs may progress in some cases to a large decalcification with cavitation.^4,43
^ The appearance of WSLs or cavitation after fixed appliance removal is discouraging.^27^ Although the chance of developing WSLs is highly increased with prolonged treatment, studies have proved that these lesions may develop after the first month of bonding the appliance in cases of deteriorated oral hygiene.^50^ The rapid occurrence rate declares the necessity for the utilization of effective preventive strategies from the early beginning of treatment. Improvement of oral hygiene regimen, professional prophylactic cleaning with fluoride application, and the development of fluoride-releasing orthodontic materials are considered early methods to prevent WSL occurrence.^43^ Incorporating different nanoparticles within orthodontic primer bonding agents or orthodontic adhesives and hydrogels was thoroughly studied after the evolution of nanotechnology.^2,21,29,42,46
^ One of the most interesting treatment options is the use of hydroxyapatite nanoparticles (HANPs), which mimic enamel remineralization.^28,45
^ HANPs are biologically compatible molecules with extreme affinity to enamel. Besides that, HANPs have improved anti-inflammatory properties.^7,31
^


Amelogenin, the most abundant protein in forming enamel, constitutes over 90% of the extracellular matrix (ECM).^15^ A splice variant, leucine-rich amelogenin peptide (LRAP), retains the functional N- and C-terminal domains essential for interactions with hydroxyapatite (HAP) and has demonstrated comparable efficacy to full-length amelogenin in promoting enamel remineralization.^12,20
^ However, despite its critical role, amelogenin-deficient mice still exhibit some mineralized enamel, indicating that non-amelogenin proteins, such as enamelin and ameloblastin, also contribute significantly to enamel mineralization.^48^ Recent findings suggest that amelogenin alone is a weak nucleator, but its activity is enhanced in the presence of enamelin. ^18,26
^ The great attention paid to generative dentistry led to biomimetic remineralization of enamel using synthetic amelogenin peptides.^19^ Self-assembling peptides form a three-dimensional network that aids in the remineralization of WSLs.^40,47
^ The short hydrophilic part within their molecular design enables the assembly of the peptide, which attracts calcium and phosphate from saliva, leading to the formation and arrangement of hydroxyapatite particles in a similar way to natural enamel formation.^16^ Due to its desirable properties, chitosan hydrogel has been used in the prevention and remineralization experiments alone or in combination with other materials.^32,46
^ In the present study, chitosan-based hydrogels were loaded with amelogenin peptide, and the second was loaded with HANPs, utilized in the form of dual injectable hydrogels to acquire the peptide properties in the organization, arrangement, and binding of HANPs to prevent the development of WSLs during acid attacks.

## Methods and Materials

### Ethical Approval and Informed Consent

Ethical approval for the use of extracted human teeth was obtained from the College of Dentistry, University of Baghdad, Institutional Ethics Committee (Project No. 628422, Reference No. 628; approval date: June 2, 2022). The teeth were extracted for orthodontic reasons as part of routine clinical care, with no additional procedures performed for research purposes. All patient identifiers were removed prior to sample collection. Due to the use of anonymized biological material that would otherwise be discarded, the requirement for written informed consent was waived by the Ethics Committee.

### Physicochemical Characterization of Materials

#### Amelogenin peptide

The synthetic amelogenin-derived peptide (LRAP-based analog) was obtained from CHINAPEPTIDES (Shanghai, China). The peptide had a purity of ~95.13% (HPLC verified), and the sequence provided by the manufacturer was (MPLPSYEVLTPLKWPSTDKTKREEVD), consistent with previously published functional amelogenin motifs used for enamel remineralization applications.^32^ The peptide was supplied as a lyophilized powder and reconstituted in the chitosan hydrogel at 0.5 mg/ml under sterile conditions.

#### Hydroxyapatite nanoparticles (HANPs)

The size of hydroxyapatite nanoparticles is about 13 nm (an average size of 10–12 nm, and most of the particle sizes are within 8–16 nm, rod-shaped morphology, Ca/P ratio approximately 1.67) were incorporated into the chitosan matrix at 10% w/v. Particle size and morphology specifications were provided by the manufacturer and are consistent with typical nanocrystalline hydroxyapatite used in dental biomineralization research. The nanoparticles were dispersed homogeneously into the chitosan medium using magnetic stirring to avoid agglomeration.^46^


#### Hydrogel rheology and stability

The chitosan-based hydrogel (2% w/v, medium molecular weight, 75–85% deacetylation) exhibits shear-thinning behavior and stable viscosity suitable for controlled dental application. The hydrogel remained stable during the entire study period, with no observable phase separation, precipitation, or degradation under storage at 4°C.^46^


#### Dual-syringe delivery system

A custom 3D-printed dual-injector device was fabricated to permit simultaneous co-delivery of peptide-loaded and HANP-loaded hydrogel components. The system consisted of two parallel 1 ml barrels connected to a unified applicator tip to ensure equal-volume extrusion of both hydrogels. A schematic diagram of the system has been illustrated in Figure 1.

**Fig 1a and b Fig1aandb:**
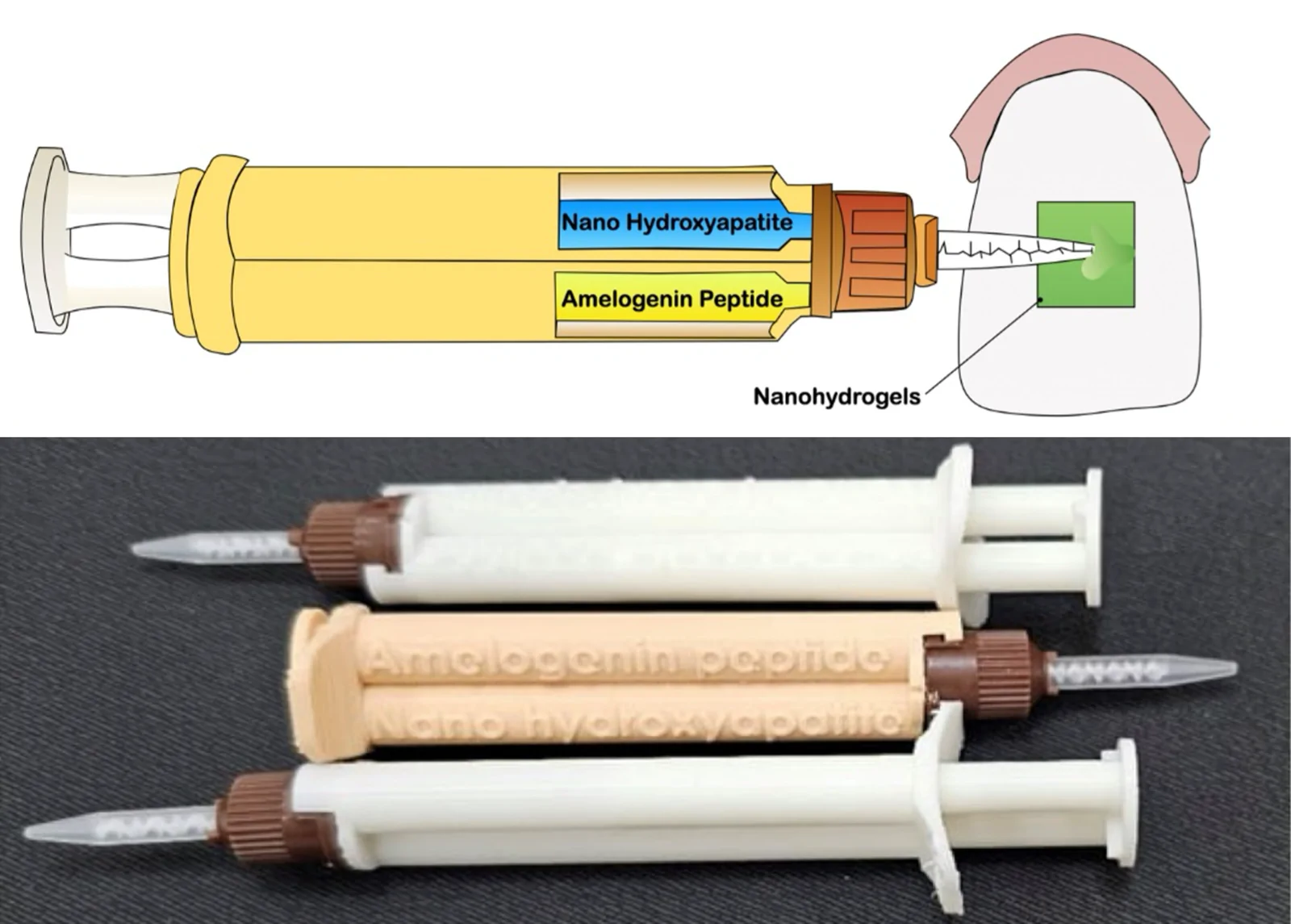
The dual-syringe delivery system. (a) Schematic illustration of the dual syringe consisting of two separate internal canals containing amelogenin peptide hydrogel and nano-hydroxyapatite hydrogel. Both components are delivered simultaneously through a single dispensing head using a unified plunger mechanism. (b) Custom 3D-printed dual-injector devices.

### Sample Size Calculation

The primary outcome of the study was defined as the change in enamel surface microhardness (ΔVHN) after pH cycling between the baseline and post-cycling measurements among the three study groups. The sample size calculation was performed using G*Power 3.1; *a priori*, based on this primary outcome, using an α error of 0.05, a β error of 0.20 (power = 80%), and an assumed common standard deviation of 1.1 VHN, derived from previous work on enamel remineralization using peptide-based or chitosan-based systems.^32^ A minimum detectable difference considered clinically relevant between groups was set, yielding an effect size compatible with a medium-to-large magnitude. Under these assumptions, the required sample size was calculated as n = 12 specimens per group (total n = 36) for a one-way comparison of three groups.

### Preparation and Storage of Enamel Samples

Human maxillary first premolar teeth extracted for orthodontic purposes were collected for this study. Immediately after extraction, teeth were rinsed with distilled water to remove blood and debris and stored in distilled water at room temperature to prevent dehydration until specimen preparation. No chemical disinfectants or preservatives were applied to avoid alteration of enamel properties. The collected teeth were examined under ×10 magnification using a stereomicroscope. The exclusion criteria included carious teeth, teeth with cracks or structural irregularities, and teeth showing visible signs of previous professional chemical treatment. Out of 280 examined teeth, 36 intact teeth were selected. The crowns were embedded in acrylic using a custom-made mold after root separation with a diamond-coated disk under continuous water cooling. On the exposed enamel surface, a 4 × 4 mm window was created on the buccal surface using acid-resistant nail varnish. Prior to baseline measurements, specimens were stored at 37°C in artificial saliva (pH 7.0) to simulate oral conditions. The artificial saliva was replenished every 24 h throughout the study period.

### Randomization and Blinding

The specimens (n = 36) were randomly assigned into three groups using a computer-generated randomization table to ensure unbiased allocation (12 teeth per group) (https://www.random.org/). All laboratory outcome measurements were performed by an examiner who was blinded to the group assignments throughout the study to minimize detection bias.

### Interventions

Baseline assessments were carried out for all specimens, which included assessment of surface microhardness (ΔVHN), fluorescence analysis, X-ray diffraction, scanning electron microscopy, and energy-dispersive X-ray spectroscopic analysis. After baseline assessment, the specimens (n = 36) were divided into three groups, each containing 12 samples. The first group was the control group and received no treatment, while the other two groups were the experimental groups in which one was subjected to surface treatment by a chitosan-based hydrogel loaded with peptide and considered as the Chi-Pep group, while the other group was treated with the simultaneous application of two chitosan-based hydrogels, one loaded with amelogenin peptide and the other loaded with hydroxyapatite nanoparticles, using a dual-syringe delivery system (Chi-Pep+HANPs group).

### Procedure

The dental specimens were stored in artificial saliva at 37°C and pH 7.0 for 21 h daily to simulate oral remineralizing conditions. Each day, specimens were removed from the artificial saliva, gently air-dried, and treated with the assigned materials for 5 min. For the Chi-Pep group, 50 µL of peptide-loaded hydrogel was applied using a calibrated micropipette. For the Chi-Pep+HANPs group, 50 µL of hydrogel was delivered using the custom-designed dual-syringe system to ensure simultaneous application. One drop was applied to the enamel surface, which was previously measured with a pipette (50 µL). The remineralizing hydrogels were applied to specimens placed on a flat surface without agitation or mechanical movement to ensure consistent material deposition and uniform experimental conditions across all specimens. The control group received no surface treatment.

At the end of 5 min, specimens were transferred directly to the demineralizing solution without intermediate rinsing with distilled water, in order to preserve the activity of the applied remineralizing agents. The specimens were then immersed in the demineralizing solution for the remaining 3 h daily at 37°C. The demineralizing buffer was made of 2 mM calcium chloride (CaCl_2_), 2 mM potassium dihydrogen phosphate (KH_2_PO_4_), 50 mM sodium acetate, and 0.879 ml acetic acid at pH 4.6 at 37°C.^33^ The pH-cycling protocol was selected to assess the preventive potential of the tested materials rather than to induce fully developed white spot lesions prior to treatment. The specimens were subjected to pH cycling for 14 days before the second assessment. After that, a reassessment of the samples was undertaken.^49^


### Measurement of Surface Microhardness (ΔVHN)

Microhardness (ΔVHN) was measured using a Vickers hardness tester (Bareiss Prüfgerätebau, Oberdischingen, Germany) with a load force of 100 g and 15 s dwell time of 15 s; three indentations were made at about 100 µm spacing. The resulting indentations were scanned with a 400× microscopic lens, and an average was calculated.^11^


### Fluorescence Analysis

At an excitation wavelength of 365 nm, the fluorescence spectra were collected using the FL WinLab™ software with a PerkinElmer LS 55 (Waltham, MA, USA) luminescence spectrometer.^10^


### X-Ray Diffraction (XRD)

The XRD pattern for samples at the baseline and after pH cycling was recorded with the aid of an X’Pert Pro diffractometer (Philips, Cambridge, UK), with monochromatized Cu(Kα) radiation, 40 kV, and 44 mA, with a sampling step size of 0.08 and a 2θ range of 5–65 degrees. The mineral phase of the samples, the average crystallite size, and the dislocation density were calculated.

The particle sizes were obtained by the Debye–Scherrer equation as follows:

D = 0.9λ cos / B cos θ_(hkl)_, where:

D: Crystallite size,λ: Wavelength of the X-ray beam,B: Full width at half maximum of the peak at the maximum intensity, andθ_(hkl)_: Peak diffraction angle.^38^


The dislocation density is inversely related to the square of the crystal size as follows: ρ* *= 1/D^2^, where ρ is the dislocation density, and D is the crystallite size.^54^


### Scanning Electron Microscopy (SEM) and Energy-Dispersive X-Ray Spectroscopy (EDX)

After sputtering the specimens with an Au coating, an environmental scanning electron microscope (ESEM, Quanta 200, FEI Company, USA) was used to evaluate the surface characteristics of the specimens at baseline and after pH cycling. A silicon drift detector at 20 kV was used to evaluate the mineral phase. Calcium (Ca) wt%, phosphate (P) wt%, and Ca/P ratio were calculated. All the specimens were evaluated with five magnifications: ×400, ×700, ×2000, ×5000, and ×50,000.^34^


### Statistical Analysis

All statistical analyses were carried out using the SPSS software package (version 25, SPSS, Chicago, USA). The primary outcome was the change in enamel surface microhardness (ΔVHN) after pH cycling. Secondary outcomes included fluorescence measurements, XRD parameters (crystal size and dislocation density), and Ca wt%, P wt%, and Ca/P ratio from EDX analysis. Data were first assessed for normality using the Shapiro–Wilk test. Homogeneity of variance among groups was evaluated using Levene’s test. As the data met the assumptions for parametric testing for within-group comparisons between baseline and post-cycling measurements, a paired-samples t-test was used. Inter-group differences at baseline and after pH cycling were analyzed using one-way ANOVA. When a significant main effect was found, Tukey’s honestly significant difference (HSD) post hoc test was applied to control the family-wise error rate for multiple pairwise comparisons between groups. Effect sizes for ANOVA were expressed as partial eta squared (η_p_
^2^), and effect sizes for paired comparisons were accompanied by 95% confidence intervals (95% CI) for the mean differences. The threshold of statistical significance was set at *p* < 0.05.

## Results

### Microhardness (ΔVHN)

The descriptive statistics of enamel microhardness before and after intervention are illustrated in Figure 2. At baseline, the highest mean microhardness value was related to the Chi-Pep+HANPs group, followed by the Chi-Pep and Control groups, respectively. However, the difference was less than one hardness number. After pH cycling, microhardness values declined for the Control group, and there was a smaller amount of decline for the Chi-Pep group. Meanwhile, the Chi-Pep+HANPs group showed an improved mean hardness value.

**Fig 2 Fig2:**
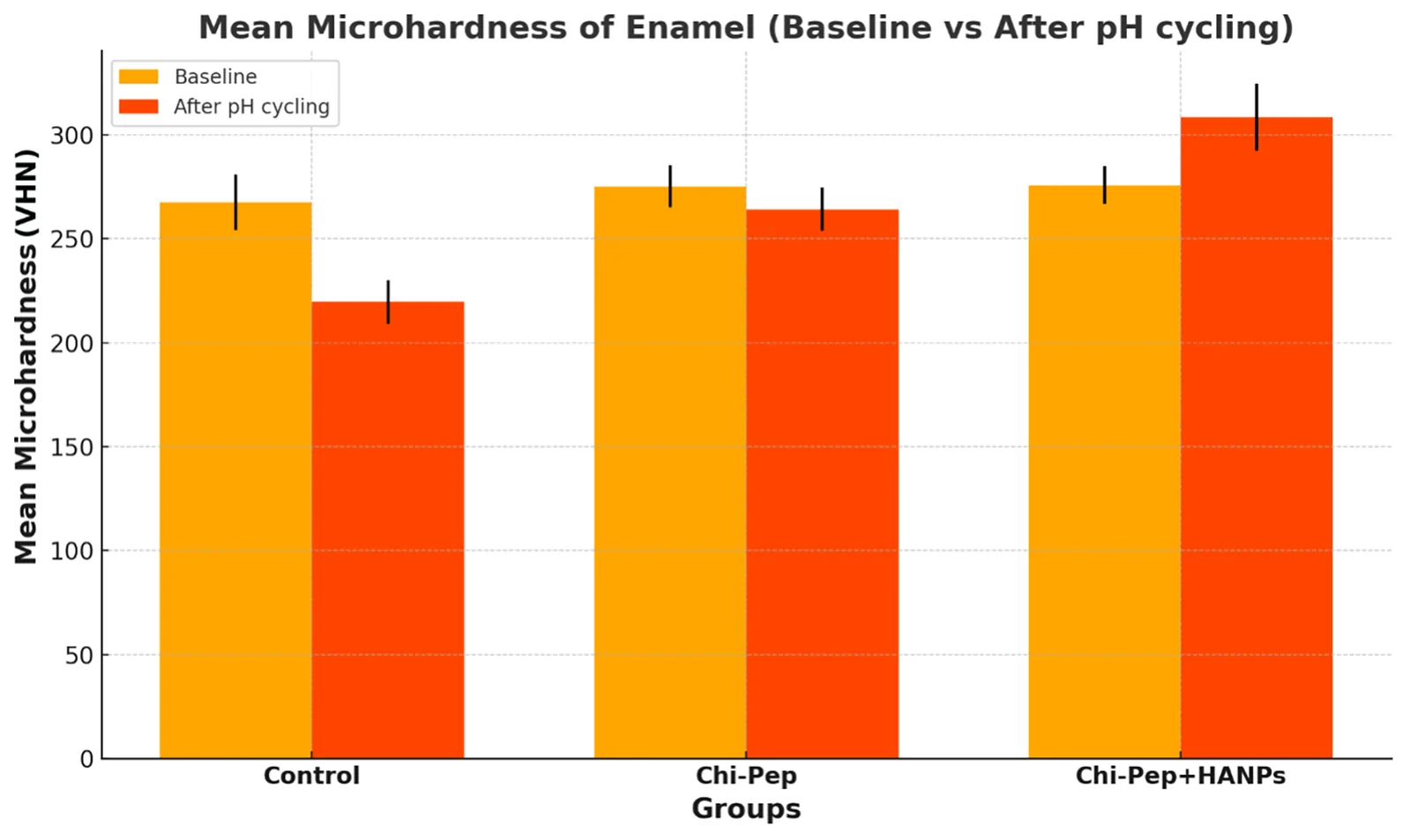
Descriptive statistics of enamel microhardness at baseline and after pH cycling. The maximum mean microhardness value at baseline was related to the Chi-Pep+HANPs group. After pH cycling, the Chi-Pep+HANPs group revealed an improved mean hardness value.

Paired t-test for all three groups compared the baseline and after pH cycling microhardness values for each group. The result revealed statistically significant differences for all groups (Table 1). Figure 1s (supplementary materials) illustrates the error bars of the microhardness values at the baseline and after pH cycling at a 95% confidence interval.

**Table 1 Table1:** Paired t-test for microhardness (ΔVHN) between experimental groups

Groups		Mean	SE	95% CI		*p* value
				Lower	Upper	
Control	Baseline – after pH cycling	47.917	5.383	36.069	59.765	0.001*
Chi-Pep	Baseline – after pH cycling	10.833	3.620	2.866	18.801	0.012*
Chi-Pep+HANPs	Baseline – after pH cycling	−32.583	8.537	−51.374	−13.793	0.003*
*The difference is significant at *p* < 0.05.						

One-way ANOVA revealed no statistically significant difference in baseline microhardness among the three groups (Table 2), F (2, 33) = 0.178, *p* = 0.838, η_p_
^2^ = 0.011, indicating a negligible effect size. After pH cycling, a statistically significant difference in microhardness was observed among groups, F (2, 33) = 12.547, *p* = 0.001, η_p_
^2^ = 0.432, indicating a large effect size.

**Table 2 Table2:** One-way ANOVA of microhardness with partial eta squared (η_p_
^2^)

Condition	SS between	SS within	SS total	*F*	*p* value	Partial eta squared (η_p_ ^2^)*
Baseline	510.056	47,315.583	47,825.639	0.178	0.838	0.011 (small)
After pH cycling	47,348.667	62,268.083	109,616.750	12.547	0.001	0.432 (large)
SS: sum of squares. *Interpretation of η_p_ ^2^ values: 0.01 = small, 0.06 = medium, 0.14 = large.						

Levene test of homogeneity revealed a homogeneous distribution of data after pH cycling, so Tukey HSD post-hoc multiple comparisons after pH cycling (Fig 3) showed statistically significant differences among all groups.

**Fig 3 Fig3:**
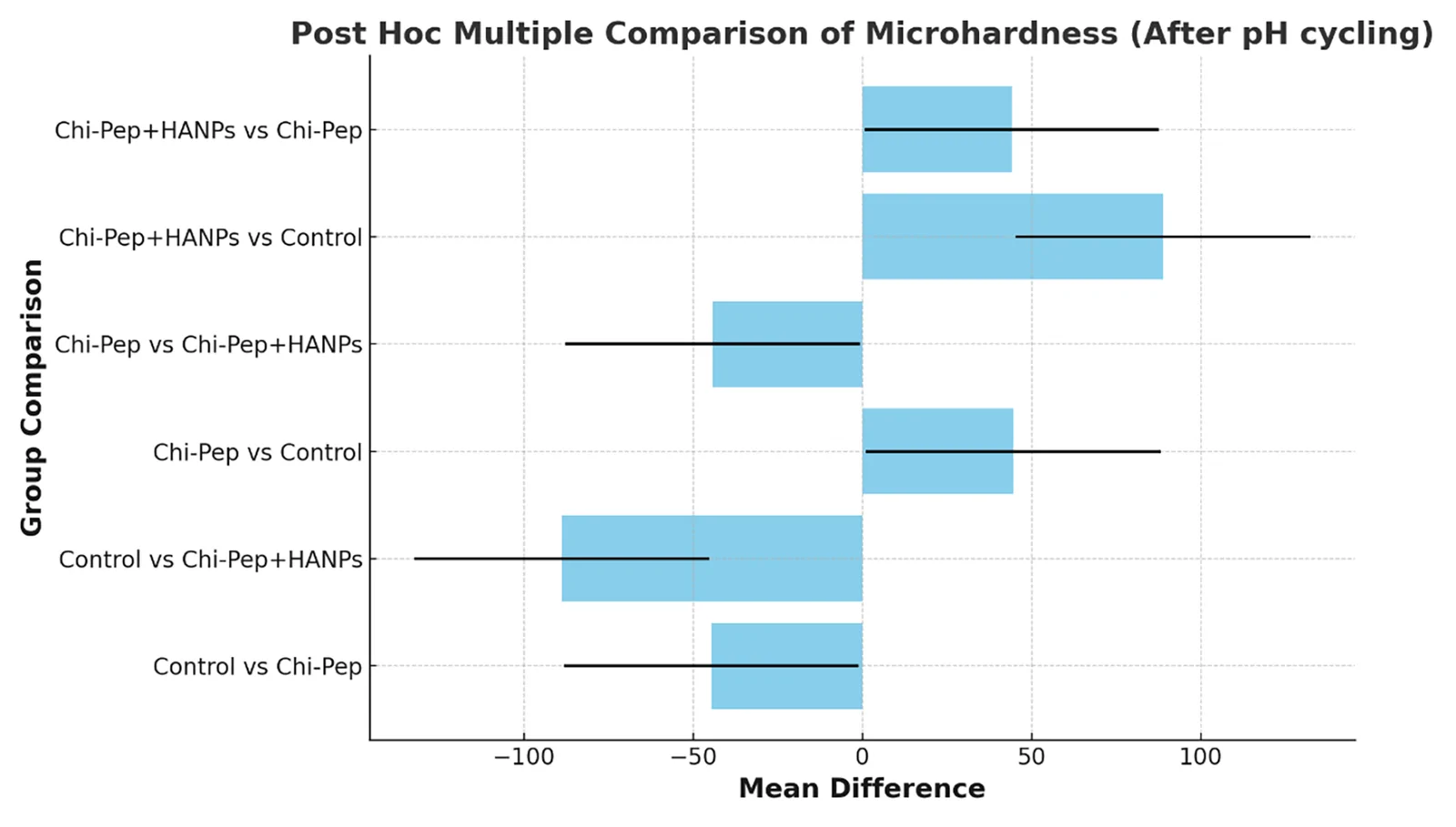
Tukey HSD post hoc multiple comparisons of enamel microhardness after pH cycling. Statistically significant differences were observed among all groups.

### Fluorescence Spectroscopy

The overall fluorescence curves at the baseline and after pH cycling with different surface treatments are shown in Figure 4. At 365 nm excitation wavelength, the emission spectra were observed in the region between 440–500 nm. An obvious reduction in fluorescence was seen in the control group. The Chi-Pep-treated group showed no changes in fluorescence. Chi-Pep+HANPs-treated group showed improved fluorescence.

**Fig 4 Fig4:**
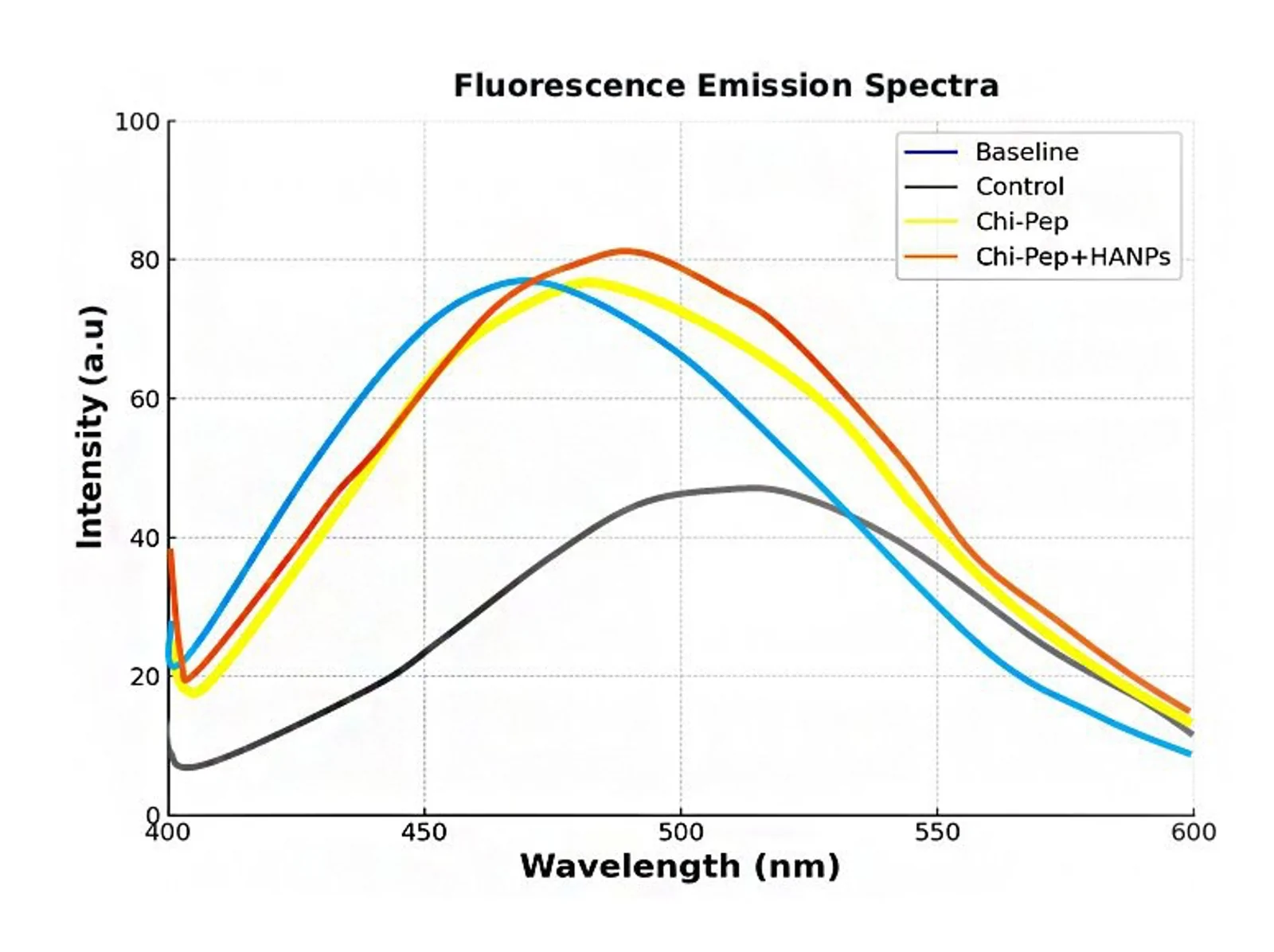
Emission spectra showed fluorescence reduction in the control group. The Chi-Pep group showed no changes in fluorescence. The Chi-Pep+HANPs group showed improved fluorescence.

### X-Ray Diffractometry

The XRD patterns of all samples were compared with the standard card (Reference code: 01-072-1243). Figure 2s (supplementary materials) illustrates that the diffraction peaks were comparable to those of hydroxyapatite. For sound enamel, distinct peaks were observed for (002) at 2θ = 25.875 and (211) at 2θ = 31.741, which reflect the crystallinity of hydroxyapatite particles. After pH cycling, the Chi-Pep+HANPs-treated group showed the strongest peaks. The Chi-Pep and control groups revealed a broadening of these peaks, which is related to hydroxyapatite particle destruction. The average particle size before pH cycling for intact enamel was 27.4 nm, as shown in Table 3.

**Table 3 Table3:** Crystal size and dislocation density of the study groups

Groups		Crystal size (nm)	Dislocation (nm^−2^)	Dislocation (nm^−2^) × 10^−3^
Baseline		27.4	0.001331984	1.331
After pH cycling	Control	21.7	0.002123638	2.123
	Chi-Pep	22.08	0.002051171	2.051
	Chi-Pep+HANPs	28.9	0.001197304	1.197


After pH cycling, the particle size for the control and Chi-Pep groups was reduced to 21.7 nm and 22.08 nm, respectively. The Chi-Pep+HANPs group showed improvement with an average crystal size of 28.8 nm. The inverse relation between particle size and dislocation is illustrated in Figure 5.

**Fig 5 Fig5:**
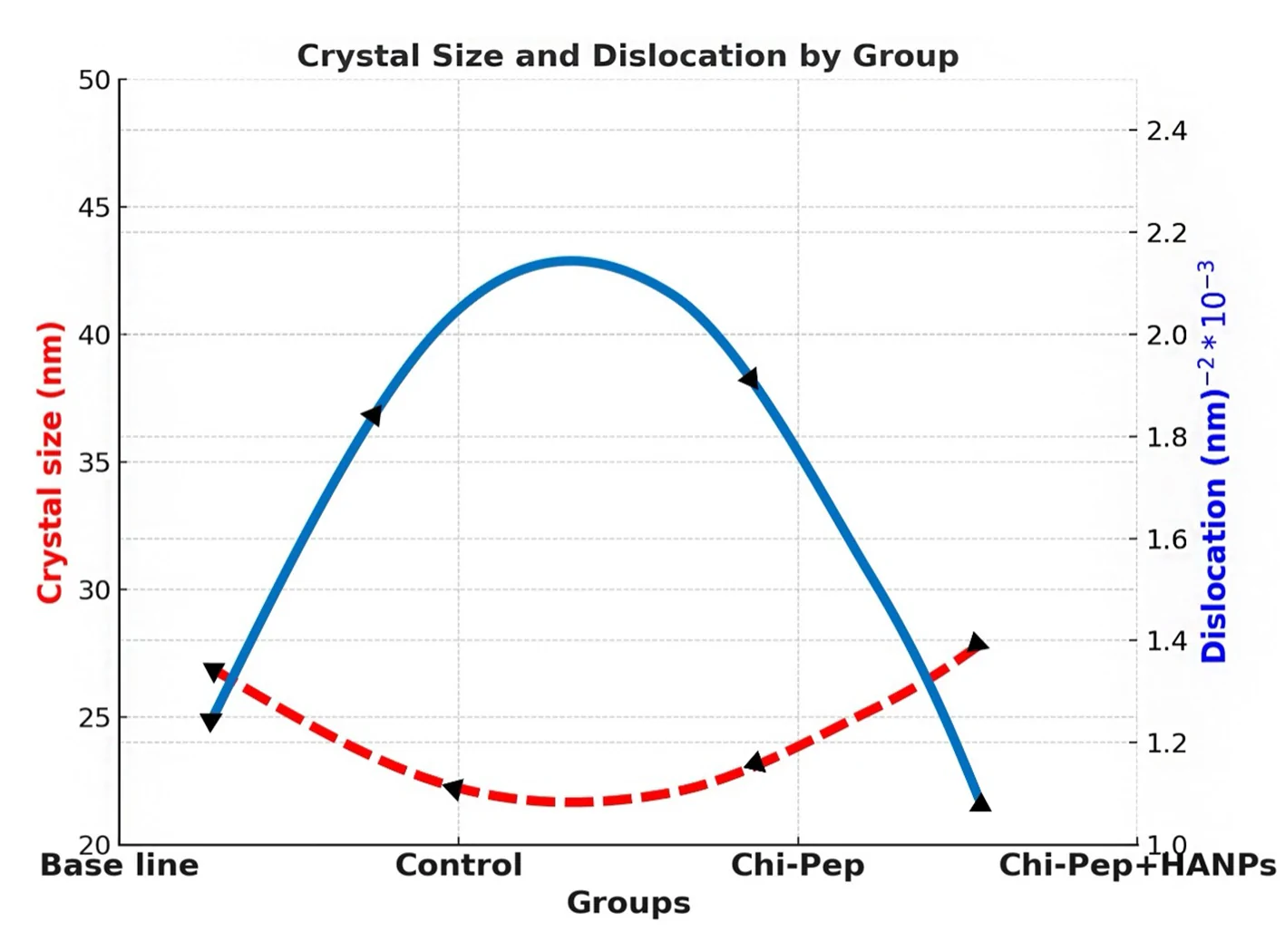
The correlation between dislocation density and crystallite size. As the particle size increases, the dislocation density declines.

### Energy-Dispersive X-Ray Spectroscopy

The descriptive statistics of Ca wt%, P wt%, and Ca/P ratio of the experimental groups at the baseline and after pH cycling are illustrated in Figure 3s.

A paired t-test was performed for each experimental group to compare Ca wt%, P wt%, and Ca/P ratio at the baseline and after pH cycling (Table 4). For all groups, there was a statistically significant difference. However, for the Control and Chi-Pep groups, the differences were reductions in the values of Ca wt%, P wt%, and Ca/P ratio. For the Chi-Pep+HANPs group, the results revealed a statistically significant elevation in the values of Ca wt%, P wt%, and Ca/P ratio.

**Table 4 Table4:** Paired t-test of Ca wt%, P wt%, and Ca/P ratio in all groups

		Groups								
		Control			Chi-Pep			Chi-Pep+HANPs		
		Mean difference	SE	*p* value	Mean difference	SE	*p* value	Mean difference	SE	*p* value
Ca wt%	Baseline – after pH cycling	30.230	1.409	< 0.001*	2.120	0.735	0.015*	−1.800	0.704	0.027*
P wt%	Baseline – after pH cycling	11.552	1.039	< 0.001*	2.598	0.520	< 0.001*	−1.473	0.252	< 0.001*
Ca/P ratio	Baseline – after pH cycling	0.2320	0.100	0.024*	−0.135	0.054	0.030*	0.051	0.028	0.100
*The difference is significant at *p* < 0.05.										

At baseline, no statistically significant difference in Ca wt% was detected, F (2, 33) = 0.940, *p* = 0.401, η_p_
^2^ = 0.054. Whereas, after pH cycling, a highly significant difference in Ca wt% was observed among groups, F (2, 33) = 593.131, *p* < 0.001, η_p_
^2^ = 0.973, demonstrating an extremely large effect size. Baseline P wt% did not differ significantly among groups, F (2, 33) = 1.073, *p* = 0.354, η_p_
^2^ = 0.061. A statistically significant difference in P wt% was found after pH cycling, F (2, 33) = 30.951, *p* < 0.001, η_p_
^2^ = 0.652, indicating a large effect size. No statistically significant difference in Ca/P ratio was observed at baseline, F (2, 33) = 0.738, *p* = 0.486, η_p_
^2^ = 0.043. Following pH cycling, a statistically significant difference in Ca/P ratio was detected among groups, F (2, 33) = 5.505, *p* = 0.009, η_p_
^2^ = 0.250, reflecting a large effect size, as shown in Table 5, and Figure 4s.

**Table 5 Table5:** One-way ANOVA of Ca wt%, P wt%, and Ca/P ratio with eta squared (η_p_
^2^)

Variable	Condition	SS between	SS total	*F*	*p* value	Eta squared (η_p_ ^2^)*
Ca wt%	Baseline	23.145	429.479	0.940	0.401	0.054 (small)
	After pH cycling	8025.097	8248.343	593.131	< 0.001	0.973 (very large)
P wt%	Baseline	23.043	377.360	1.073	0.354	0.061 (medium)
	After pH cycling	776.176	1189.961	30.951	< 0.001	0.652 (large)
Ca/P ratio	Baseline	0.144	3.367	0.738	0.486	0.043 (small)
	After pH cycling	1.559	6.233	5.505	0.009	0.250 (large)
SS: sum of squares. *Interpretation of η_p_ ^2^ values: 0.01 = small, 0.06 = medium, 0.14 = large.						

Tukey HSD post hoc multiple comparisons (Fig 5s) revealed no statistically significant differences for Ca wt%, P wt%, and Ca/P ratio for all groups at the baseline. After pH cycling, statistically significant differences were observed for all readings between the Control and Chi-Pep+HANPs groups. Between the Control and Chi-Pep+HANPs groups, the Ca/P ratio showed statistically significant differences. However, no statistically significant differences were observed when comparing the Ca/P ratio of Control and Chi-Pep groups after pH cycling.

### Scanning Electron Microscopy

The microscopic presentation of the sound enamel surface showed a smooth, homogeneous surface with a minor degree of scratches. With ×50,000 magnification, densely packed hydroxyapatite crystals can be observed. After exposure to an acidic environment through pH cycling, the control group showed an outer enamel surface with lost surface integrity. The empirical lines appear due to the dissolution of the prism core, and the honeycombed pattern is now evident. With high magnification, gross enamel porosities could be observed (Fig 6s). Peptide application before pH cycling results in the preservation of the enamel surface with a very small amount of mineral dissolution and micropores when compared with the control group (Fig 7s). Surface treatment with peptides and HANPs simultaneously resulted in the preservation of the enamel surface. Microscopic presentation with low magnification showed a smooth, intact enamel surface. With higher magnification, the regular “fish scale” texture of enamel prisms appeared. With very high magnification, the presentation of densely packed hydroxyapatite particles is still evident (Fig 8s).

**Fig 6 Fig6:**
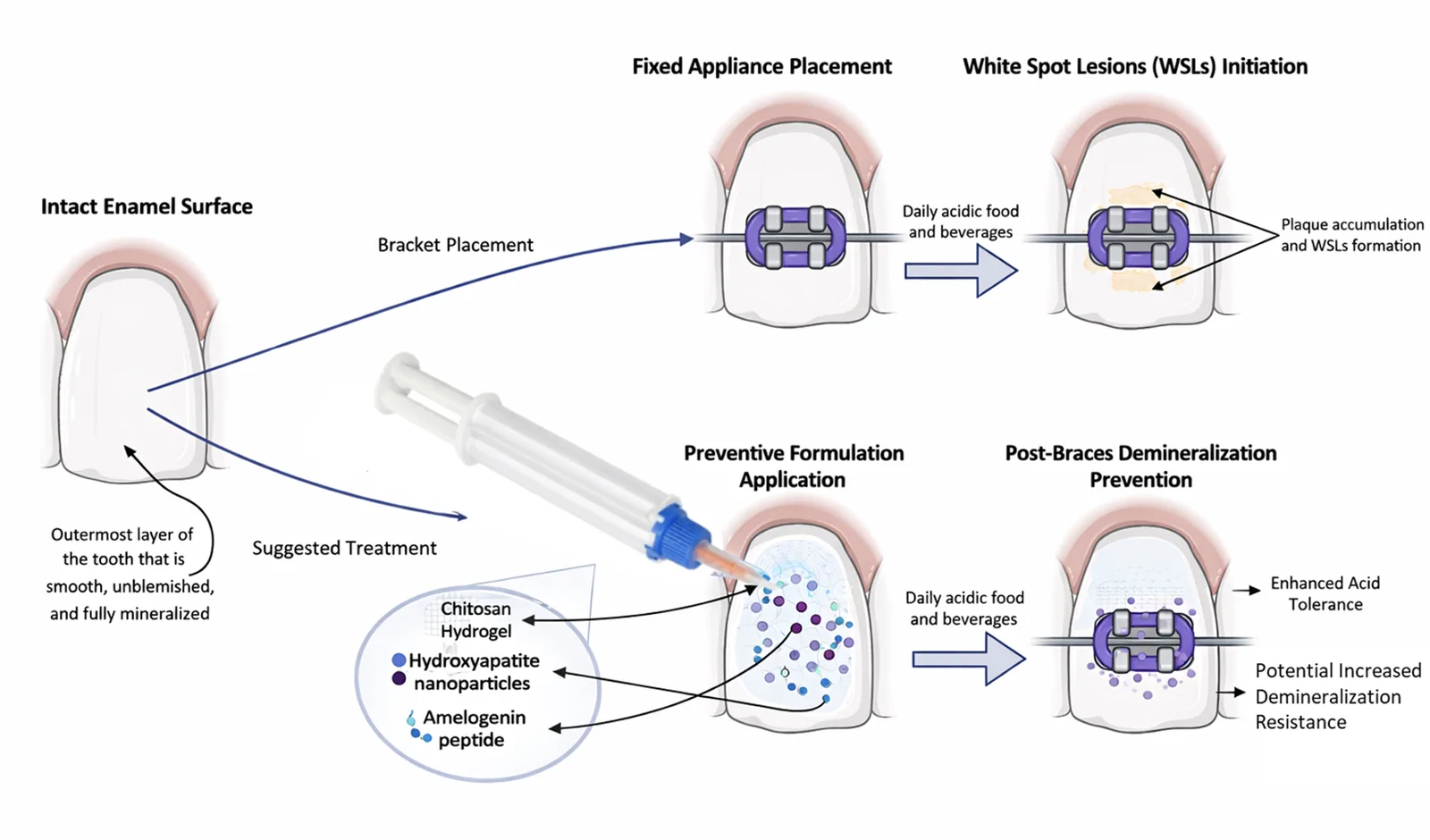
Illustration of a preventive approach for white spot lesion (WSL) management during orthodontic treatment. This figure outlines a stepwise strategy to prevent WSLs during orthodontic treatment. Orthodontic brackets increase the risk of plaque accumulation and acid exposure, leading to enamel demineralization and WSL formation. To combat this, a biomimetic hydrogel containing amelogenin peptides, hydroxyapatite nanoparticles (HANPs), and chitosan is applied. This formulation enhances remineralization, protects against acid attacks, and preserves enamel health around brackets, reducing the risk of WSLs (created in BioRender. Review, A. (2024) https://BioRender.com/g23m768).

## Discussion

Bonded fixed orthodontic appliances provide a favorable oral environment for bacterial plaque accumulation, which consequently leads to WSL formation.^6^ WSLs mainly develop on the buccogingival surfaces, considered one among several adverse effects of orthodontic treatment.^43^ Preventing the occurrence of this adverse effect of orthodontic treatment becomes mandatory in highly susceptible patients.^23^ Although several studies were conducted to evaluate the effect of variable methods and materials on the remineralization of WSLs during fixed orthodontic treatment, no ideal solution for this adverse effect has been developed.^50^ In response to this need, the present study aimed to develop a nanohydrogel that combines amelogenin peptide with hydroxyapatite nanoparticles (HANPs) within a chitosan matrix, providing an efficient and easy application method to prevent WSLs (Fig 6). To mimic the oral environment, pH cycling was used to evaluate the preventive potential of this formulation under acidic conditions. The present study aims to develop a nanohydrogel with an efficient and easy application method to prevent the occurrence of WSLs.^37^ For the evaluation of the preventive effect, pH cycling was performed to reflect the oral environment. Chitosan has been suggested as a preventive therapeutic material and has shown a reduction in bacterial biofilm and preserved the mineral composition of the enamel surface.^14^ In this study, the addition of HANPs to the peptide hydrogel led to a statistically significant enhancement in the enamel surface’s mechanical characteristics, surpassing the effects of peptide-only treatment.

Although large effect sizes (η^2^) were observed, these values should be interpreted with caution, as they may be influenced by controlled *in vitro* conditions and sample homogeneity. Therefore, the magnitude of the effect should be considered within the context of the experimental model.

The zeta potential of the amelogenin peptide/chitosan hydrogel showed a stable hydrogel with a zeta potential value of 36.7 mV, while adding HANPs reduces zeta potential, confirming their binding with the peptide.^46^ This result is comparable to previous work on the effect of toothpaste containing HANPs, which has the potential to prevent WSLs during orthodontic treatment with fixed appliances.^22^ A previous study has demonstrated that HANPs primarily act as biomimetic building blocks for enamel repair and mineral deposition, rather than as potent antimicrobial agents, unless chemically modified or doped with metallic ions.^35^ Besides that, treating the enamel surface with HANPs before orthodontic bonding showed a preventive effect.^8^ The biomimetic nature of HANPs, which imitate natural enamel, is instrumental in these findings. HANPs can enter subsurface enamel porosities, creating a scaffold for ion exchange that aids in organizing HANPs within interprismatic spaces, enhancing enamel microhardness values and resistance to acid exposure.^17^ This effect suggests that HANPs not only strengthen the enamel but also restore its natural resilience. A previous study on the effect of HANPs containing gel on enamel remineralization stated that after demineralization, the remnant of protein created a scaffold for ion exchange, which aided in the arrangement of HANPs into interprismatic subsurface enamel porosities, leading to enhanced enamel microhardness values.^44^ The fluorescence of the enamel surface treated with the peptide was almost that of sound, untreated enamel. Fluorescence serves as a marker for enamel health, indicating mineral composition and structural integrity. The combined peptide and HANP treatment achieved fluorescence levels closer to healthy enamel than the control or peptide-only groups, suggesting improved mineralization and restoration potential.^41^ Amelogenin’s role as a scaffold during natural enamel formation has particular relevance for WSL prevention. Its ability to attract calcium and phosphate ions in a manner similar to natural enamel processes aids in the remineralization of enamel surfaces exposed to acid, suggesting a strong preventive mechanism against WSLs.^36^


However, adding HANPs with peptides resulted in better improvement. The result of this study comes in line with previous literature in which it was concluded that self-assembling peptides, when combined with inorganic remineralizing analogs, enhance enamel remineralization.^5,24
^ However, no previous work was conducted to evaluate the effect of amelogenin peptide on the prevention of WSLs. In XRD, the (002) peaks indicated the formation of HANPs, which are compatible with that of natural enamel, in which the newly growing minerals were arranged along the tooth axis and perpendicular to the dentinoenamel junction.^26^ The Chi-Pep+HANPs group showed the most intense peaks with an increase in particle size when compared with the baseline sound enamel surface. This result indicated that treating the enamel surface with amelogenin peptide combined with HANPs promotes a preventive effect with the improvement of surface characteristics. This result was supported by the result of a previous study in which XRD analysis revealed that the amelogenin peptide participates in the growth of a crystal layer comparable to that of sound enamel. They concluded that the arrangement of the new crystals along the C axis depended on the existence of the amelogenin peptides.^34^ The increased particle size of enamel hydroxyapatite promotes a lower dislocation rate and greater resistance to acid attacks.^53^


This result aligns with previous studies indicating that amelogenin peptides contribute to the crystal growth of enamel-like structures along the C axis, suggesting that peptide presence is essential for arranging new crystals in a manner that mimics natural enamel. By increasing crystal size,^13^ HANPs effectively reduce dislocation rates and enhance resistance to acid attack. Larger hydroxyapatite crystals are typically associated with a lower rate of acid dissolution, highlighting the functional benefit of combining peptides with HANPs for optimal enamel protection. The result of the present study illustrated that the size of HANPs was greater in the Chi-Pep+HANPs-treated group when compared with other groups, with minimal dislocation density.^55^ A reduced dislocation density implies a more stable crystal structure and further enhances the enamel’s resistance to demineralization. This increase in particle size and reduction in dislocation density underscores the potential for peptide and HANPs treatment to reinforce enamel at the molecular level, creating a robust barrier against acidic challenges. To evaluate the enamel surface morphology, five magnification levels were evaluated. Previous works confirmed enhanced enamel architecture after remineralization with amelogenin peptides or HANPs.^3,25
^ The same was observed in the results of this study, where adding amelogenin peptide alone, or simultaneously with HANPs, resulted in the preservation of the enamel surface against acid attack. EDX analysis of the chemical compositions of enamel revealed an elevation in the calcium and phosphate weight percent in the Chi-Pep+HANPs treatment, which indicates greater resistance to demineralization.^51^ The same elevation was observed previously when the demineralized enamel was treated with amelogenin peptide.^34^ The elevated Ca and P levels support that HANPs, in combination with peptides, provide a mineral-rich surface similar to sound enamel, further enhancing its ability to withstand acidic challenges. This finding suggests that incorporating HANPs and peptides could be a potent strategy for promoting enamel remineralization, particularly in high-risk orthodontic patients prone to WSL formation.

## Conclusion

The present study demonstrated that the application of amelogenin peptide in conjunction with hydroxyapatite nanoparticle-loaded chitosan hydrogels preserved and improved the structural and chemical characteristics of enamel surfaces exposed to acid challenges *in vitro* using a pH-cycling model. These findings suggest a potential preventive effect *in vitro* and support further investigation of this biomimetic approach. However, the clinical relevance of these results remains to be established, and future *in vivo* and clinical studies, including evaluation of orthodontic bracket bond strength, are required before clinical application can be considered. This *in vitro* design did not include a chitosan-only negative control or a fluoride-based positive control, which would allow clearer differentiation of the carrier effect and comparison with gold-standard preventive agents. Future studies incorporating such controls are recommended to strengthen clinical translation.

### Acknowledgments

#### Clinical relevance

The clinical relevance of using nano peptide hydrogel on extracted premolars with artificially induced white spot lesions (WSLs) lies in its potential to enhance enamel remineralization, prevent caries progression, and improve esthetic outcomes. By delivering bioactive peptides at the nanoscale, it promotes mineral deposition, repairs enamel, and offers a non-invasive, biocompatible treatment option. This approach could reduce the need for invasive restorative procedures, making it a promising tool in preventive dentistry, though further clinical studies are needed to confirm its long-term efficacy.

## References
